# Haem relieves hyperoxia-mediated inhibition of HMEC-1 cell proliferation, migration and angiogenesis by inhibiting BACH1 expression

**DOI:** 10.1186/s12886-021-01866-x

**Published:** 2021-02-25

**Authors:** Lan Jian, Yang Mei, Chen Xing, Yuan Rongdi

**Affiliations:** 1 Department of Ophthalmology, Xinqiao Hospital, Army Medical University, Xinqiao Road, Shapingba District, Chongqing, 400032 China; 2 Department of Army Occupational Disease, State Key Laboratory of Trauma, Burn and Combined Injury, Daping Hospital, Army Medical University, Chongqing, 400042 China

**Keywords:** BTB and CNC homology l, Haem, Microvascular endothelial cells, Retinopathy of prematurity, Vascular endothelial growth factor

## Abstract

**Background:**

Hyperoxia-mediated inhibition of vascular endothelial growth factor (VEGF) in the retina is the main cause of impeded angiogenesis during phase I retinopathy of prematurity (ROP). Human retinal angiogenesis involves the proliferation, migration and vessel-forming ability of microvascular endothelial cells. Previous studies have confirmed that BTB and CNC homology l (BACH1) can inhibit VEGF and angiogenesis, while haem can specifically degrade BACH1. However, the effect of haem on endothelial cells and ROP remains unknown.

**Methods:**

In this report, we established a model of the relative hyperoxia of phase I ROP by subjecting human microvascular endothelial cells (HMEC-1) to 40% hyperoxia. Haem was added, and its effects on the growth and viability of HMEC-1 cells were evaluated. Cell counting kit 8 (CCK8) and 5-ethynyl-2′-deox-yuridine (EdU) assays were used to detect proliferation, whereas a wound healing assay and Matrigel cultures were used to detect the migration and vessel-forming ability, respectively. Western blot (WB) and immunofluorescence (IF) assays were used to detect the relative protein levels of BACH1 and VEGF.

**Results:**

HMEC-1 cells could absorb extracellular haem under normoxic or hyperoxic conditions. The proliferation, migration and angiogenesis abilities of HMEC-1 cells were inhibited under hyperoxia. Moderate levels of haem can promote endothelial cell proliferation, while 20 μM haem could inhibit BACH1 expression, promote VEGF expression, and relieve the inhibition of proliferation, migration and angiogenesis in HMEC-1 cells induced by hyperoxia.

**Conclusions:**

Haem (20 μM) can relieve hyperoxia-induced inhibition of VEGF activity in HMEC-1 cells by inhibiting BACH1 and may be a potential medicine for overcoming stunted retinal angiogenesis induced by relative hyperoxia in phase I ROP.

**Supplementary Information:**

The online version contains supplementary material available at 10.1186/s12886-021-01866-x.

## Background

Retinopathy of prematurity (ROP) is a retinal vascular disease in premature and low-birth-weight infants and is one of the main causes of blindness in children [1]. With the development of perinatal medicine and neonatology and the establishment of neonatal intensive care units (NICUs), the survival rate of premature and low-birth-weight infants has gradually improved, and the incidence of ROP has shown an obvious upward trend. The main pathological features of ROP are stunted retinal angiogenesis in the early stage and pathological neovascularization in the later stage. The main cause of stunted angiogenesis in early ROP is the relative hyperoxia owing to infants leaving the uterus prematurely and receiving oxygen therapy, which inhibits VEGF expression in the retina. Early angiogenesis development determines the severity of retinal hypoxia in the later stage, which ultimately defines the extent of retinal neovascularization [2, 3]. To solve the problem of neonatal hypoxia and reduce mortality, oxygen therapy is sometimes necessary [4]; however, there is no effective intervention for retinopathy caused by relative hyperoxia in early ROP.

BTB and CNC homology l (BACH1) is an important transcription factor involved in the regulation of cellular reactive oxygen species, haem homeostasis, haematopoiesis and immunity [5]. It can inhibit VEGF expression and angiogenesis by inhibiting haem oxygenase-1 (HO-1) activity, enhancing the production of mitochondrial reactive oxygen species in endothelial cells, and competitively inhibiting β-catenin [6–8]. Previous studies have shown that haem can specifically inhibit BACH1 expression in mice without affecting normal physiological functions. This activity has also been confirmed in triple-negative breast cancer (TNBC) cells and murine erythroleukemia (MEL) cells [9, 10].

However, the effect of haem on endothelial cells and ROP has not been reported. In this report, we established a model of relative hyperoxia during early ROP by exposing HMEC-1 cells to 40% hyperoxia. Then, the effects of different doses of haem on the proliferation, migration and angiogenesis of HMEC-1 cells were observed, and the protein expression of BACH1 and VEGF was quantified. The results showed that the proliferation, migration and angiogenesis of HMEC-1 cells were inhibited under hyperoxia, and 20 μM haem promoted VEGF expression by inhibiting BACH1 expression in HMEC-1 cells and relieved the hyperoxia-induced inhibitory effects on angiogenesis. These findings provide a certain experimental basis for haem as a potential medicine for the treatment of stunted angiogenesis induced by relative hyperoxia during the early stages of ROP.

## Methods

### HMEC-1 cell culture

HMEC-1 cells were purchased from American Type Culture Collection (ATCC, Rockefeller of Maryland), and the certificate and STR test results are supplemented in Additional files 1 and 2. According to the previous protocol established by our research group [11], cells were cultured in MCDB131 medium containing 10% foetal bovine serum (FBS, Gibco, USA), 1% antibiotics (including streptomycin 0.1 mg/mL and penicillin 100 U/mL, Gibco), 2 mM glutamine (25,030, Gibco) and 1 μg/mL hydrocortisone (M3451, Abmole, USA) [12]. A working solution of 10 mM haem (51,280, Sigma, USA) was prepared in 20 mM NaOH (diluted in PBS) [9]. HMEC-1 cells were cultured in vitro and divided into normoxia and hyperoxia groups. The normoxia group was maintained in an incubator containing 5% CO_2_, 21% O_2_ and 74% N_2_, and the hyperoxia group was maintained in an incubator containing 5% CO_2_, 40% O_2_ and 55% N_2_. Both groups were incubated at 37 °C and 90% humidity [13, 14]. Meanwhile, medium (blank), vehicle (20 mM NaOH; negative control) or different concentrations of haem were added into the culture medium according to groups. The cells used in the experiment were from passages 5 to 10.

### High-performance liquid chromatography (HPLC)

#### Sample preparation

As described in previous methods [15, 16], HMEC-1 cells were seeded in 6-well plates (1 × 10^5^/well) for 12 h under normoxic conditions to allow adherence to the well bottom. The culture medium was changed, and the cells were incubated under normoxic or hyperoxic conditions and treated according to the designated group (0 μM haem, 20 μM haem or vehicle) for 48 h (the medium was changed every 24 h). Next, cells were washed twice with PBS, scraped with a cell curettage in 500 μL of PBS per well, and centrifuged at 300×g for 5 min. After the supernatant was removed, 200 μL of Tris EDTA buffer (Tris: 10 mM, EDTA: 1 mM, pH: 7.4) was added per sample, which was shaken for 1 h, and ultrasonicated for 5 min. Then, 600 μL of HPLC-grade acetonitrile (100,269, Honeywell, Korea) was added to the samples before they were vortexed and centrifuged at 2500×g for 5 min. Upon removal of the supernatant, 200 μL of Tris EDTA buffer was added to the pellet before 800 μL of acetonitrile:1.7 M HCl (8:2, v/v) was added dropwise and shaken for 20 min to extract haem from the protein fraction into the acetonitrile. To create a two-phase liquid system, 200 μL of saturated MgSO_4_ and 0.02 g of NaCl were added to the samples, which were vortexed for 5 min and centrifuged at 2500×g for 5 min before 200 μL was taken from the top organic layer for HPLC. A blank and a standard used for quantification purposes were subjected to the same extraction and clean-up steps to rule out any contamination during clean-up and to calculate the yield.

#### Test method

First, a standard curve was made with purified haem at concentrations of 1, 5, 10, 20, 50, and 100 μM. Then, 20 μL of the prepared sample was added to the detection bottle and subjected to HPLC (Waters, e2695, USA) with the following parameters: wavelength, 190–700 nm; chromatographic column, C18 reverse column; column temperature, 25 °C; injection volume, 10 μL; mobile phase A, 0.1% trifluoroacetic acid (TFA) solution; mobile phase B, 0.1% TFA-acetonitrile; flow rate, 1 mL/min; and elution gradient: 0–10 min, A 95–40%, B 5–60%; 10–20 min, A 40%, B 60%; and 20–25 min, A 40–95%, B 60–95%. The peak area of haem content was obtained. Three samples were taken from each group, and the experiment was repeated three times.

### CCK8 assay

Cell proliferation was measured using CCK-8 (42,830, MCE, USA) according to the supplier’s protocol. Cells were seeded in 96-well plates (5 × 10^3^/well) and then allowed to adhere to the well bottom after 12 h under normoxic conditions. In the dose effect test, the cells were treated with different haem concentrations (9 groups: 0, 5, 10, 20, 40, 80, 160, 320 μM and negative control) and then cultured under normoxic or hyperoxic conditions for 48 h (the medium was changed every 24 h). Subsequently, 100 μL of medium containing CCK-8 reagent (1:10 dilution) was added to each well (including a blank control) and incubated at 37 °C for 1 h. Finally, the absorbance at a wavelength of 450 nm of each well was measured on a microplate reader (Gene Company Limited, ELX800ux, Hong Kong) [17]. The cell proliferation value was calculated as follows: OD_450_ = OD_group_ - OD_blank_. In the time effect test, 4 groups were established: normoxia control group (21% O2 + 0 μM haem), normoxia haem group (21% O2 + 20 μM haem), hyperoxia control group (40% O2 + 0 μM haem) and hyperoxia haem group (40% O2 + 20 μM haem), OD_450_ values were measured by the CCK8 method at 0, 12, 24, 48, 72 and 96 h initial treatment. Four replicate wells per condition were measured in each experiment, and the experiment was repeated three times.

### EdU assay

Cell proliferation activity was also analysed by the EdU assay (RiboBio, Guangzhou, China) according to the manufacturer’s instructions. Cells were seeded in Millicell plates (1.5 × 10^4^/well, 200 μL) for 12 h and allowed to adhere to the well bottom under normoxic conditions. Cells were then treated with the indicated haem concentration (0 μM haem, 20 μM haem or vehicle) and cultured under normoxic or hyperoxic conditions for 48 h (the medium was changed every 24 h). Then, 200 μL of reagent A (1:1000) was added to the cells and incubated for 2 h at 37 °C before cells were fixed with 4% paraformaldehyde for 30 min, destained with 2 mg/mL glycine decolorization for 5 min, washed with PBS for 5 min, and permeabilized with 0.5% Triton for 10 min. The other reagents (B, C, D, E and F) were mixed together according to the instructions. Finally, the cells were washed with PBS for 5 min, stained with DAPI (1:1000, 4083, CST, USA) for 5 min, washed with PBS and sealed with an anti-fluorescence quenching solution [18]. Three regions per well were randomly selected and imaged under a laser confocal microscope (Leica, SP8, Germany), and the number of proliferating cells was counted by ImageJ software (National Institutes of Health, Bethesda, Germany). Each experiment was repeated at least three times.

### Wound healing assay

HMEC-1 cells were seeded in 6-well plates (1 × 10^5^/well) under normoxic conditions, and the medium was changed every 24 h until the cells reached confluency. The monolayer was then scratched with a 200-μL pipette tip and washed with PBS. The cells were treated as indicated (0 μM haem, 20 μM haem or vehicle) and cultured under normoxic or hyperoxic conditions. The central area of the scratch in the 6-well plate was identified under an inverted phase contrast microscope (Zeiss, Primo Vert, Germany) equipped with a 5× objective and CCD cameras; photos were taken at 0, 12 and 24 h after scratching [19]. After the photos were taken, drawing software (Photoshop 8.0, Adobe, USA) was used to first draw a solid line along both sides of the scratched area on the photo taken at 0 h and dotted lines along the cell migration front on the photos taken at 12 and 24 h after scratching. The scratch area was calculated by ImageJ software. The area between solid lines is S_0h Scratch area_, and the area between dotted lines is S_12/24h Scratch area_, S_Migration area_ = S_0h Scratch area_ - S_12/24h Scratch area_. The experiment was repeated independently three times.

### Tube formation assay

Tube formation was assessed as previously described [20]. HMEC-1 cells were seeded in 6-well plates (1 × 10^5^/well) for 12 h and then allowed to adhere to the well bottom under normoxic conditions. The cells were treated as indicated (0 μM haem, 20 μM haem or vehicle) and cultured under normoxic or hyperoxic conditions for 48 h (the medium was changed every 24 h). Matrigel (356,234, Corning, USA) was added to precooled Millicell plates (200 μL/well) and polymerized for 40 min at 37 °C. Then, cells in each treatment group were digested by trypsin and resuspended to a density of 3.5 × 10^5^ cells/mL in the corresponding medium. Finally, the cell suspensions (200 μL/well) were added to Millicell plates containing Matrigel. The cells were incubated for 4 h under normoxic or hyperoxic conditions, washed once with Hank’s balanced salt solution (HBSS), and treated with 8 μg/mL calcein AM fluorescent dye (HBSS dilution, 200 μL/well; 354,216, Corning) for another 4 h under normoxic or hyperoxic conditions. After the plates were washed twice with HBSS, three randomly selected regions in each well were viewed using a laser confocal microscope (Leica, SP8) and imaged. Angiotool software was used to measure the length and area as well as the number of junctions of the tubular-like structures [21]. Three independent experiments were performed.

### Western blot (WB) assay

HMEC-1 cells were seeded in 6-well plates (1 × 10^5^/well) for 12 h under normoxic conditions until they adhered to the well bottoms as previously described [22]. The cells were then subjected to the indicated treatments (0 μM haem, 20 μM haem or vehicle) and cultured under normoxic or hyperoxic conditions for 48 h (the medium was changed every 24 h). The cells were washed with PBS, incubated in 100 μL of lysis buffer for 30 min, and scraped from the well bottom with a 1-mL sterile pipette tip. Finally, the samples were collected and completely disrupted by ultrasonication for 5 min. The lysates were centrifuged at 12000×g for 15 min at 4 °C, and total protein was extracted from the supernatant. The protein concentration was detected by the double octanoic acid protein quantitative kit (Beyotime, ShangHai, China) before the sample was diluted with 2× loading buffer and boiled for 10 min. Proteins were separated by gel electrophoresis and transferred to a 0.45 μm polyvinylidene fluoride membrane. After they were blocked with 5% skim milk for 2 h, the membranes were incubated successively with primary antibodies overnight at 4 °C and secondary antibodies for 2 h at room temperature. The primary antibodies used and their respective dilutions are as follows: anti-BACH1 (1:1000, ab49657, Abcam, UK), anti-VEGF (1:1000, ab46154, Abcam), and anti-β-actin (1:10000, BS6007M, Bioworld, Minnesota); the antibodies were diluted with WB-specific diluent (Beyotime). The secondary antibodies used and their respective dilutions are as follows: goat anti-rabbit (1:5000, BS13278, Bioworld) and goat anti-mouse (1:5000, BS12478, Bioworld); both antibodies were diluted with TBST. Immunoreactive bands were developed by Immobilon Western Chemiluminescent Horseradish Peroxidase Substrate (Vilber-Lourmat, France) according to the manufacturer’s instructions. All bands were quantified by ImageJ software and normalized to the β-actin values. Three independent experiments were performed.

### Immunofluorescence (IF) assay

HMEC-1 cells were seeded in Millicell plates (1.5 × 10^4^/well, 200 μL) for 12 h and then cultured under normoxic conditions until the cells adhered to the well bottoms as previously described [11]. The cells were then subjected to the indicated treatments (0 μM haem, 20 μM haem or vehicle) and cultured under normoxic or hyperoxic conditions for 48 h (the medium was changed every 24 h). Then, the cells were washed with PBS three times for 5 min, fixed with 4% paraformaldehyde for 30 min, permeabilized with 0.3% Triton for 20 min, and blocked with 5% goat serum for 30 min. Next, the cells were treated with anti-BACH1 (1:100, ab49657, Abcam), anti-VEGF (1:250, ab1316, Abcam), or anti-PECAM-1 (1:100, sc-133,091, SANTA, USA) antibodies overnight at 4 °C. After the cells were washed with PBS three times, they were incubated for 2 h at 37 °C with goat anti-rabbit IgG/Alexa Fluor 488 (1:300, bs-0295G-AF488, CST) and goat anti-mouse IgG/Alexa Fluor 594 (1:300, bs-0296G-AF594, CST) before they were washed with PBS again and subjected to nuclear staining with DAPI (1:1000, 4038, CST) for 5 min. Finally, the cells were washed with PBS and sealed with an anti-fluorescence quenching solution. Three randomly selected regions in each well were viewed under a laser confocal microscope and imaged, and the fluorescence intensity was quantified by ImageJ software. To reduce the error, we used the automatic default value of the system when setting the threshold value. Each experiment was repeated at least three times. A negative control was set in the experiment, including the omission of the primary antibody and the use of an non-specific polyclonal or isotype-matched monoclonal primary antibody. In all cases, negative controls showed weak staining.

### Statistical analysis

SPSS 20.0 (IBM, Armonk, USA) statistical software was used for statistical analysis. All experiments were performed at least three times. The results are expressed as the means ± SD. One-way ANOVA or multivariate analysis of variance was used in cases with homogeneity of variance and normal distribution; for all other cases, a nonparametric test was used. *P* < 0.05 indicated statistical significance.

## Results

### HMEC-1 can absorb extracellular haem under normoxic or hyperoxic conditions

**Fig. 1:**
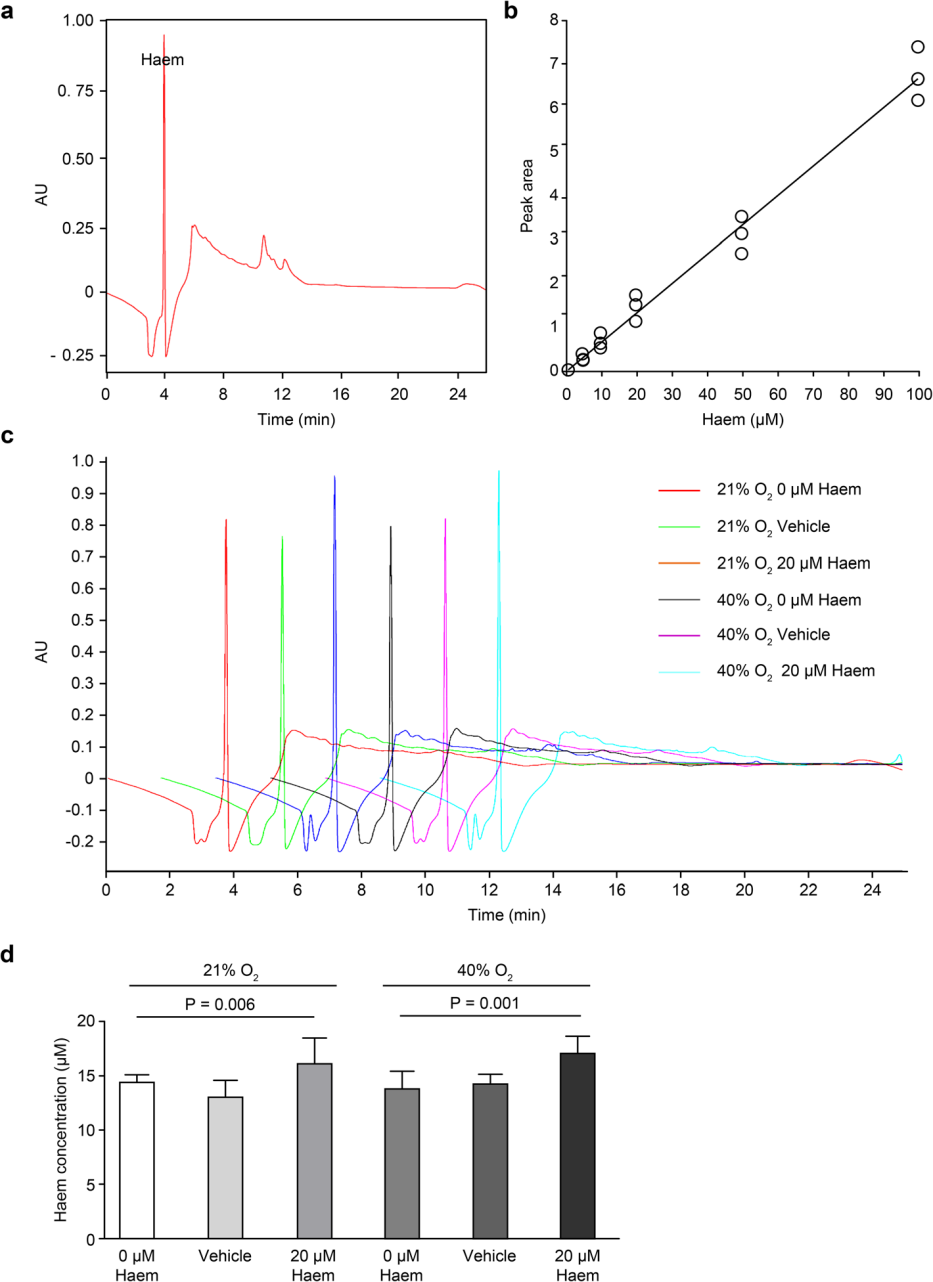
The intracellular haem concentration in HMEC-1 cells was detected by HPLC. **a**, HPLC results of purified haem. The peak that appeared at 3–4 min corresponds to the chromatographic peak of haem. **b**, A standard curve of haem at concentrations of 1, 5, 10, 20, 50 and 100 μM was established based on the peak identified in A. **c**, HPLC results of haem levels in the different treatment groups. **d**, Diagrams summarizing the quantitative analysis of the peak areas in the different treatment groups. All data are presented as the means ± SD, and the experiment was repeated three times (*n* = 3). One-way ANOVA was performed

The HPLC results of the purified haem showed that the peak that appeared at 3–4 min was the chromatographic peak of haem (Fig. 1a), and a standard curve was established according to the peak area of haem at concentrations of 1, 5, 10, 20, 50 and 100 μM (Fig. 1b). The peak area increased with increasing haem concentrations. Next, the peak area of haem content in each treatment group was analysed. The results showed that under normoxia, the peak area of the 20 μM haem group was significantly larger than that of the blank and negative control groups, and the difference was statistically significant (*P* < 0.01). Under hyperoxia, the peak area of the 20 μM haem group was significantly larger than that of the blank and negative control groups, and the difference were statistically significant (*P <* 0.01) (Fig. 1c and d). This indicates that endothelial cells can increase their haem content by absorbing extracellular haem under normoxic or hyperoxic conditions.

### Moderate levels of haem can relieve the inhibition of hyperoxia on HMEC-1 proliferation

**Fig. 2:**
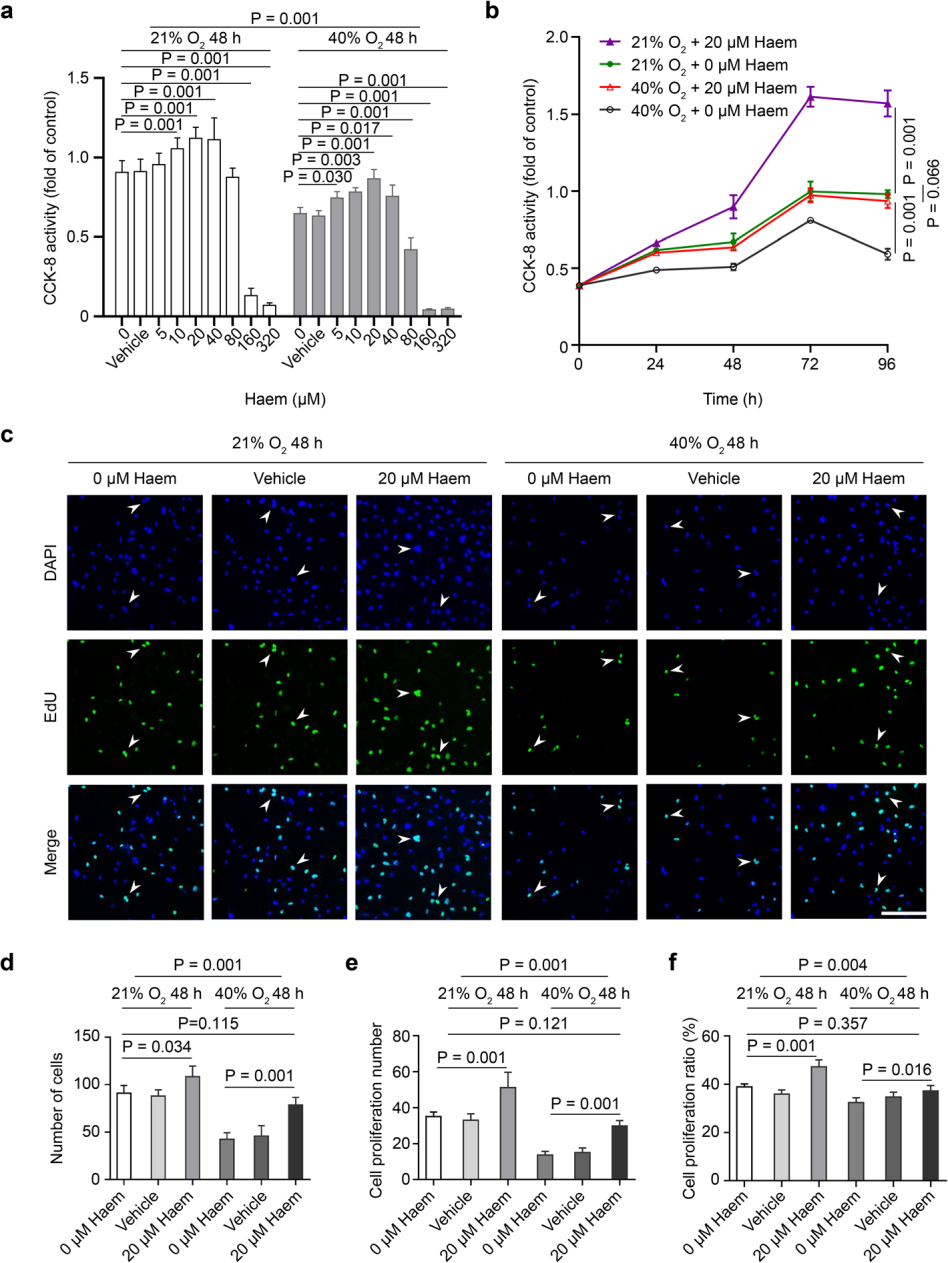
Effect of haem on HMEC-1 cell proliferation. **a**, Diagram showing the effect of different concentrations of haem on HMEC-1 cell proliferation as detected by the CCK-8 method. All data are presented as the means ± SD, and the experiment was repeated three times (*n* = 4). One-way ANOVA or multivariate ANOVA was performed. **b**, Diagram showing the effect of different incubation times of 20 μM haem on HMEC-1 cell proliferation as detected by the CCK-8 method. All data are presented as the mean ± SD, and the experiment was repeated three times (*n* = 4). Multivariate ANOVA with repeated measurements test was performed. **c**, Images representative of the proliferative activity of HMEC-1 cells as measured by the EdU assay. Under a laser confocal microscope, proliferating cells were stained green (488), and nuclei were stained blue (DAPI). The arrow indicates a representative HMEC-1 cell with proliferative activity (scale bar = 200 μm). **d-f**, Quantitative analysis of the total number of cells, the number of cells with proliferative activity and the percentage of cells with proliferative activity among HMEC-1 cells subjected to different treatments. All data are presented as the means ± SD, and the experiment was repeated three times (*n* = 3). One-way ANOVA or multivariate ANOVA was performed

Under hyperoxic conditions, HMEC-1 cell proliferation is inhibited; however, 5–40 μM haem increased the proliferative ability of HMEC-1 cells, whereas concentrations of 80 μM or greater inhibited proliferation. Under normoxic conditions, 10–40 μM haem increased the proliferative ability of HMEC-1, whereas concentrations of 160 μM or greater inhibited proliferation. Based on the results, 20 μM haem has the peak effect on HMEC-1 proliferation under normoxic and hyperoxic conditions (*P* < 0.001) (Fig. 2a). The time effect curve of HMEC-1 cell proliferation showed that HMEC-1 cells first showed increased proliferation over time and peaked at 72 h before the rates began to decrease. Under hyperoxic or normoxic conditions, the temporal proliferation curves of cells treated with 20 μM haem were higher than those of the control group (*P <* 0.001), but there was no significant difference between the 20 μM haem hyperoxia group and normoxia control group (*P* > 0.05), which indicated that 20 μM haem can relieve the inhibitory effect of hyperoxia on HMEC-1 proliferation (Fig. 2b).

The EdU assay indicates cells with proliferative activity with green fluorescence (Fig. 2c). Data analysis showed that the total number of cells, the number of cells with proliferative activity and the percentage of cells with proliferative activity decreased under hyperoxic conditions (*P* < 0.001) but increased under hyperoxic and normoxic conditions upon treatment with 20 μM haem (*P* < 0.05). There was no significant difference between the 20 μM haem hyperoxia group and the normoxic control group (*P* > 0.05), which further verified that 20 μM haem could relieve the inhibitory effect of hyperoxia on HMEC-1 cell proliferation (Fig. 2d-f).

### Haem relieves the inhibitory effect of hyperoxia on HMEC-1 migration

**Fig. 3:**
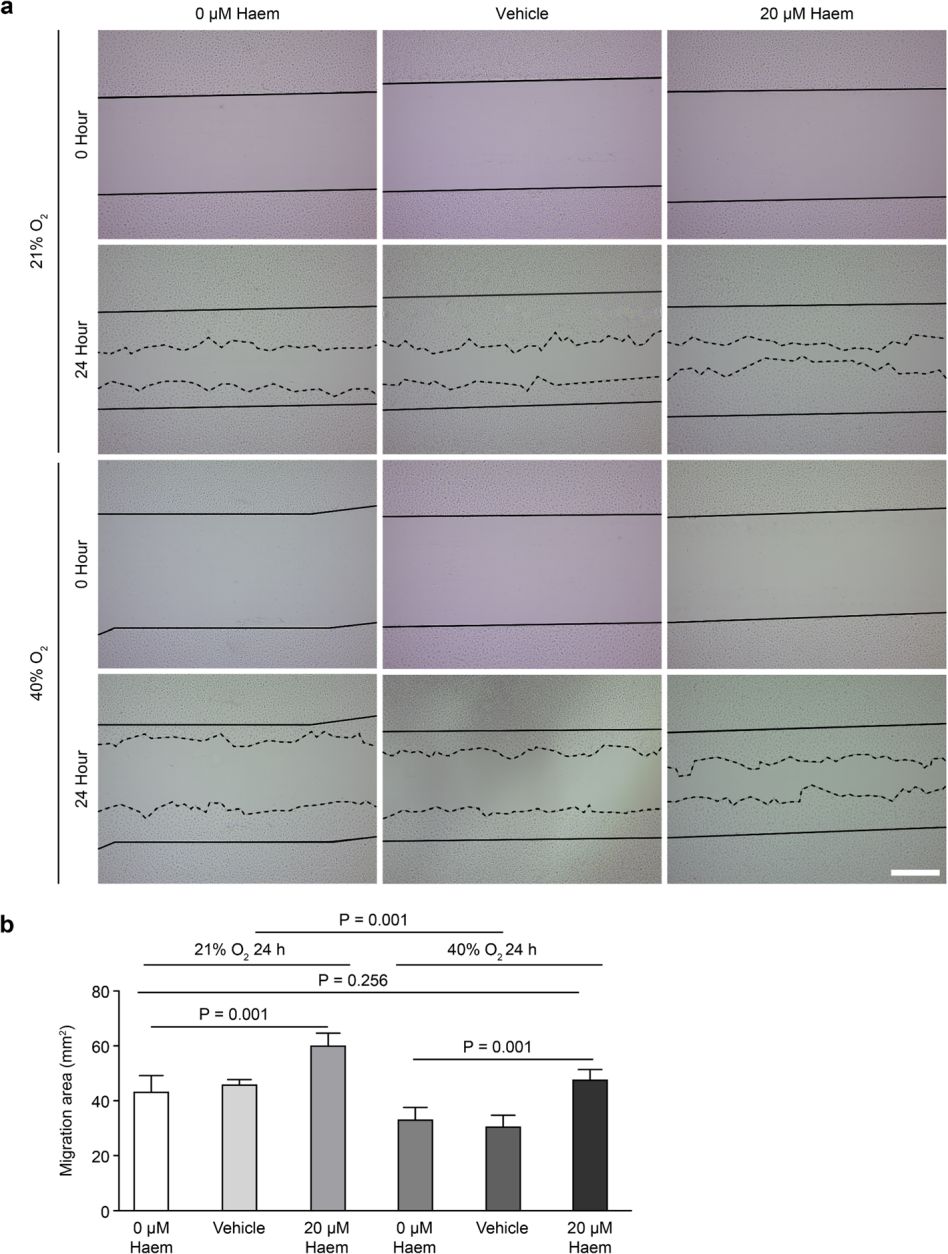
Effect of haem on HMEC-1 cell migration. **a**, Images of HMEC-1 cells were taken at 0 h and 24 h after scratching; the solid line indicates the edges of the wound at 0 h after scratching, and the dotted line indicates was the migration front of cells at 24 h after scratching (scale bar = 500 μm). **b**, Quantitative analysis of the HMEC-1 cell migration area. All data are presented as the means ± SD, and the experiment was repeated three times (*n* = 3). One-way ANOVA or multivariate ANOVA was performed

In the wound healing assay, we drew solid lines to mark the edges of the wound at 0 h after scratching and dotted lines to mark the fronts of cell migration at 24 h after scratching (Fig. 3a). ImageJ software was used to measure the scratch area, and the area between the two marked lines was the cell migration area (S_Migration area_ = S_0h Scratch area_ - S_24h Scratch area_). Data analysis showed that hyperoxia inhibited HMEC-1 cell migration (*P* < 0.001) and that 20 μM haem improved HMEC-1 cell migration under hyperoxic and normoxic conditions (*P* < 0.01). There was no significant difference between the 20 μM haem hyperoxia group and the normoxia control group (*P* > 0.05), indicating that 20 μM haem could relieve hyperoxia-induced inhibition of HMEC-1 cell migration (Fig. 3b).

### Haem relieves the inhibitory effect of hyperoxia on HMEC-1 angiogenesis

**Fig. 4:**
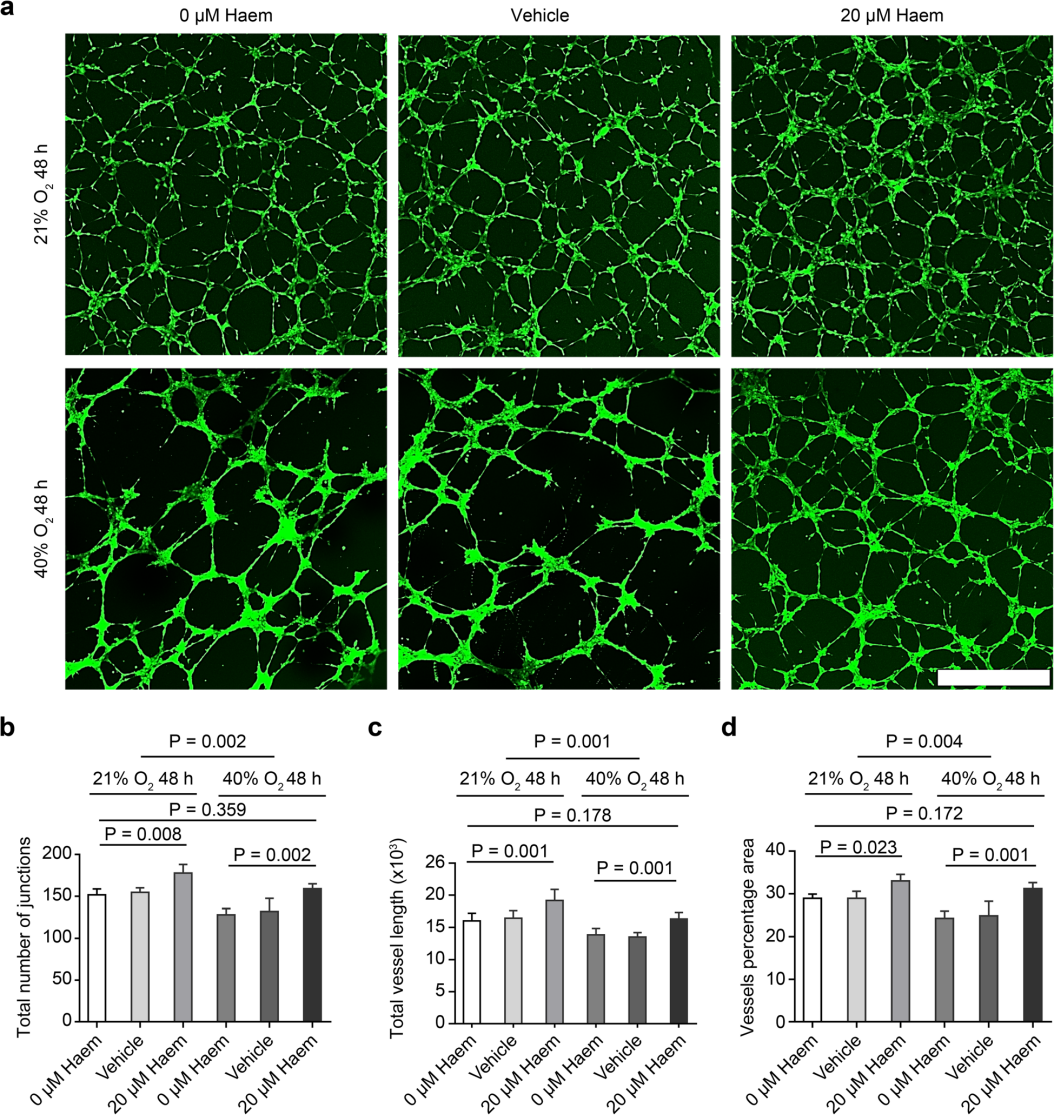
Effect of haem on the angiogenesis of HMEC-1 cells. **a**, Images of the capillary-like tube formation of HMEC-1 cells stained green (488) after 48 h of culture under different conditions. Under hyperoxic conditions alone, the vascular network was sparse, and the pore size was larger. Upon the addition of 20 μM haem, the vascular network was denser, and the pore size was finer (scale bar = 200 μM). **b-d**, Quantitative analysis of the number of junctions, length and area of capillary-like tubes formed by HMEC-1 cells. All data are presented as the means ± SD, and the experiment was repeated three times (*n* = 3). One-way ANOVA or multivariate ANOVA was performed

HMEC-1 cells were cultured with different concentrations of haem for 48 h. The capillary-like tube formation experiment shows the vascular network formed by endothelial cells with green staining. The vascular network was sparse and the pore size was larger under hyperoxic conditions compared to under normoxic conditions. After the addition of 20 μM haem, the vascular network became denser and finer (Fig. 4a). Data analysis showed that hyperoxia reduced the number of junctions and the length and area of tubes formed by HMEC-1 cells (*P* < 0.001), and 20 μM haem increased the number of junctions and the length and area of tubes formed by HMEC-1 cells under hyperoxic and normoxic conditions (*P* < 0.01). There was no significant difference between the 20 μM haem hyperoxia group and the normoxia control group (P > 0.05), which showed that 20 μM haem could relieve hyperoxia-induced inhibition of HMEC-1 cell angiogenesis (Fig. 4b-d).

### Haem (20 μM) inhibits the expression of BACH1 and promotes the expression of VEGF in HMEC-1 cells

**Fig. 5:**
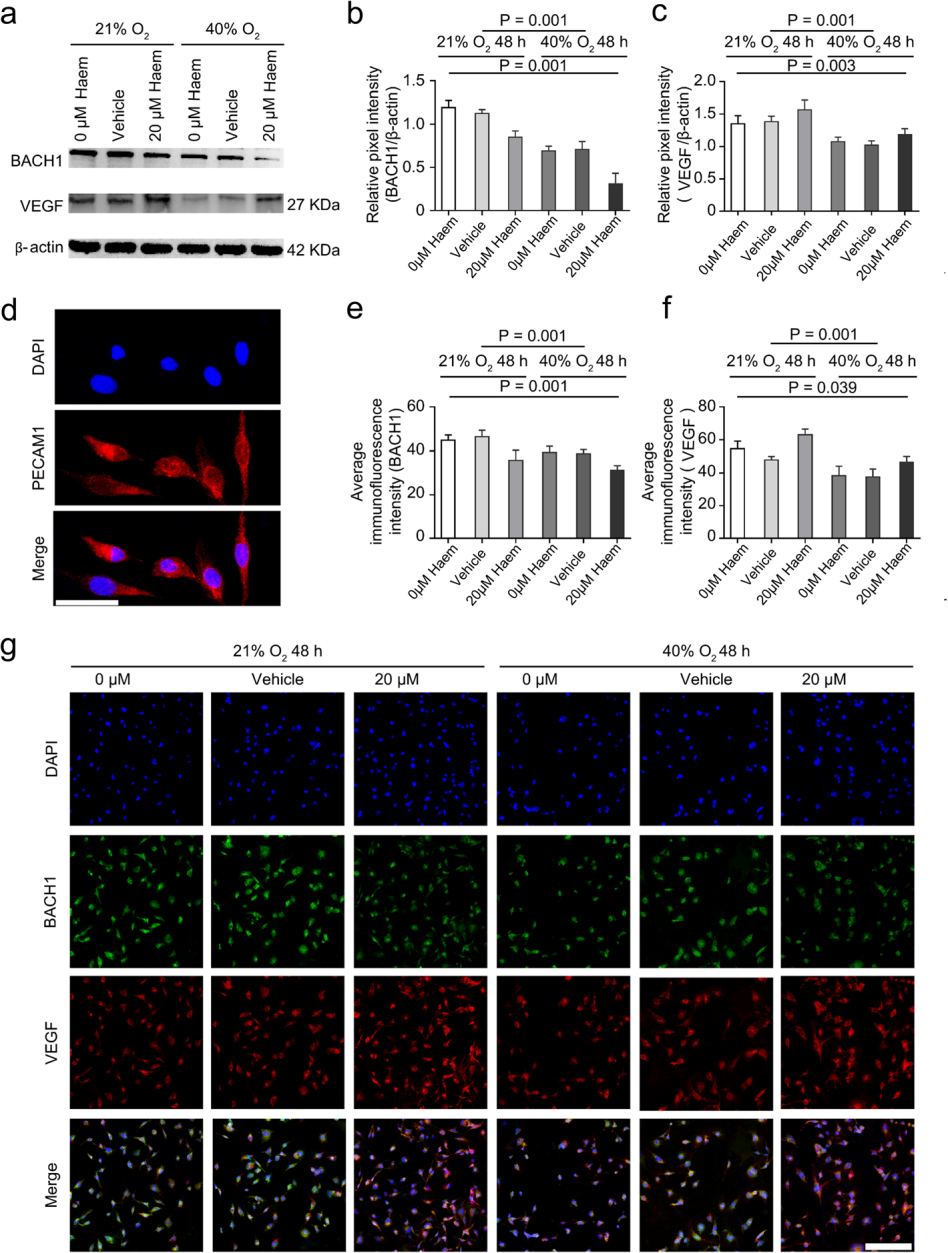
Effects of haem on the protein expression of BACH1 and VEGF in HMEC-1 cells. **a**, Western blot images of BACH1 and VEGF protein expression under hyperoxic and normoxic conditions. Upon the addition of 20 μM haem, the expression of BACH1 protein in HMEC-1 cells was lower than that in the control group whereas the expression of VEGF protein in HMEC-1 cells was higher than that in the control group. **b-c**, Quantitative analysis of BACH1 and VEGF protein expression by Western blot assay. All data are presented as the means ± SD, and the experiment was repeated three times (*n* = 3). One-way ANOVA or multivariate ANOVA was performed. **d**, The protein expression of PECAM-1 (a specific marker of endothelial cells) was stained red (594) in the cytoplasm of HMEC-1 cells. Nuclei were stained with DAPI and labelled blue (scale bar = 50 μM). **e-f**, Quantitative analysis of the fluorescence intensity per unit area of BACH1 and VEGF protein as measured by immunofluorescence. All data are presented as the means ± SD, and the experiment was repeated three times (*n* = 3). One-way ANOVA or multivariate ANOVA was performed. **g**, Immunofluorescence staining images of HMEC-1 cells. BACH1 was widely expressed in the nucleus and cytoplasm and labelled green (488), VEGF was expressed in the cytoplasm and labelled red (594), and nuclei were stained with DAPI and labelled blue (scale bar = 200 μM)

First, we identified the protein expression of platelet/endothelial cell adhesion molecule-1 (PECAM-1, also called CD31) in HMEC-1 cells by IF [23], and we found that PECAM-1 was normally expressed in the cytoplasm (Fig. 5d). Subsequently, we used IF to determine the colocalization of BACH1 and VEGF in HMEC-1 cells. The results showed that BACH1 (labelled green) was widely expressed in the nucleus and cytoplasm whereas VEGF (labelled red) was expressed in the cytoplasm (Fig. 5g). Moreover, WB (Fig. 5b and c) and IF analysis (Fig. 5e and f) revealed that hyperoxia inhibited the expression of both BACH1 and VEGF in HMEC-1 cells (*P <* 0.001). Under hyperoxic and normoxic conditions, 20 μM haem inhibited the protein expression of BACH1 (*P <* 0.01) and promoted the protein expression of VEGF in HMEC-1 cells (*P* < 0.05).

## Discussion

ROP is divided into two phases. Phase I is mainly caused by the relative hyperoxia induced by infants prematurely leaving the uterus, receiving oxygen therapy, and other factors; this leads to the rapid downregulation of the expression of vascular promoting factors such as VEGF in the retina and delayed development of retinal blood vessels to the periphery. Phase II is mainly due to the relative hypoxia induced by cessation of relative hyperoxia, stagnation of early angiogenesis, the maturation of neurons in the peripheral vascular free area of the retina, and increased energy demand, leading to overexpression of vascular promoting factors such as VEGF and consequently resulting in pathological neovascularization and eventual retinal detachment or blindness in severe cases [1]. If retinal blood vessels can form normally during phase I, the malignant proliferation of blood vessels caused by peripheral retinal ischaemia in phase II can be avoided [3]; thus, children with ROP could achieve better visual function. Studies have shown that retinal angiogenesis in humans is related to the proliferation, migration and tube-forming ability of microvascular endothelial cells [2, 24], and HMEC-1 cells are a recognized model of vascular endothelial cells [11, 25]. Therefore, we established a model of the relative hyperoxic environment of phase I ROP by subjecting HMEC-1 cells to 40% hyperoxia in vitro and found that the biological behaviours of HMEC-1 cells, such as cell proliferation, migration and tube formation, were inhibited in a 40% hyperoxic environment. We replicated some key experiments (CCK-8, EdU, tube formation) on human retinal microvascular endothelial cells (HRMECs), and the results were similar (Supplementary Fig. 1, 2).

Currently, the treatment strategy of ROP is mainly limited to phase II. Fundus laser photocoagulation and intravitreal injection of anti-VEGF drugs are the main therapies, but both have certain side effects: fundus laser treatment can cause damage to the retina and irreversible damage to children’s peripheral vision, and intravitreal injection of anti-VEGF drugs may lead to endophthalmitis and traumatic cataract. Additionally, anti-VEGF drugs can affect the development of blood vessels in other organs, especially the brain, lung and kidney, if they enter the blood circulation [3, 26]. Moreover, these two therapies are not effective in all ROP cases, and the search for therapeutic drugs that can target phase I ROP has become a hot topic in recent years. Haem is a complex of iron and tetrapyrrole protoporphyrin IX with essential functions in aerobic organisms and participates in biological oxygen transport, respiratory chain electron transfer, etc. It can be degraded to iron, carbon monoxide and biliverdin by HO-1 and has been reported to exert anti-inflammatory, antioxidant and antiapoptotic effects [27]. Haem has been used in the treatment of porphyria hepatica and is a safe and reliable drug that can be administered orally or intravenously [28], both of which are more convenient and safer than intravitreal injection. In this study, we observed that HMEC-1 cells could absorb extracellular haem, moderate haem levels can promote endothelial cell proliferation, and excessive haem can inhibit endothelial cell proliferation (Fig. 2a), which is consistent with previous reports that excess free haem is toxic via its pro-oxidant, cytotoxic and proinflammatory effects [29]. In mammals, haem homeostasis can be maintained by hemopexin neutralization or enzymatic hydrolysis of excess free haem via HO-1; up to 30 μM haem can accumulate in mitochondria that synthesize haem, while in haemolytic diseases, vascular endothelial cells may be exposed to haem concentrations as high as 100 μM before initiating a self-protective mechanism [27, 28], suggesting that 20 μM haem is a safe dose. It was reported in *Nature* that haem at a dose of 50 mg/kg could specifically degrade BACH1 in mice without affecting normal physiological functions [9]. Therefore, for the first time, we used 20 μM haem to specifically inhibit BACH1 expression in vascular endothelial cells and observed that this inhibition could promote VEGF expression in vascular endothelial cells. These findings provide a basis for administering haem to treat angiogenesis stagnation caused by the hyperoxia-induced decrease in VEGF expression in phase I ROP.

Our experimental results also showed that 20 μM haem could relieve the hyperoxia-mediated inhibition of endothelial cell proliferation, migration and tube formation ability, possibly through the mechanism of haem specifically inhibiting BACH1 to subsequently promote VEGF expression. However, when we analysed the expression of VEGF protein by WB and IF, we found that under hyperoxic and normoxic conditions, 20 μM haem could increase VEGF protein expression (*P* < 0.05) by 16.06% (WB) to 21.67% (IF) in hyperoxia and 11.91% (WB) to 15.61% (IF) in normoxia. The difference in VEGF protein expression between the 20 μM haem hyperoxia group and the normoxia control group was still statistically significant (*P <* 0.05), which indicated that 20 μM haem did not completely relieve the inhibitory effect of hyperoxia on VEGF protein expression in HMEC-1 cells. That is, 20 μM haem still did not restore VEGF protein expression in vascular endothelial cells from the simulated phase I ROP pathological environment to normal levels (Fig. 5c and f). Previous studies have shown that the occurrence and development of ROP are closely related to energy metabolism. The energy demand of retinal neurons is met by a tightly coupled vascular network. Because neurons and peripheral rod cells develop earlier than blood vessels, an energy demand is generated, which activates the physiological “hypoxia—HIF-1α—VEGF” axis—the main signalling cascade that induces angiogenesis. However, excessive nutrient deficiency and reduced oxygen supply will promote pathological neovascularization [6, 30]. By contrast, damaged retinal ganglion cells (RGCs) and photoreceptors can reduce neovascularization, and retinal blood vessels will further atrophy to match the reduced energy demand with the degeneration of neurons [31, 32]. Therefore, improving the energy metabolism efficiency of the developing retina may also be a potential target for the treatment of ROP. Recent studies have shown that inhibition of BACH1 can induce the expression of genes involved in the electron transfer chain (ETC) and promote aerobic glucose metabolism via mitochondrial respiration and the tricarboxylic acid cycle (TCA) [9]. This may be another mechanism by which haem can relieve the inhibition of HMEC-1 cell proliferation in hyperoxia. In addition, inflammation and oxygen toxicity are pathological mechanisms of ROP [3]. Interestingly, haem has been reported to exert anti-inflammatory, anti-oxidative and anti-apoptotic effects [27]; therefore, the effects of haem on endothelial cells and the pathology of ROP should also be further explored.

In conclusion, this study provides a new approach for the early prevention and treatment of stunted retinal vascular development induced by relative hyperoxia in phase I ROP. Haem can relieve hyperoxia-induced inhibition of microvascular endothelial cell proliferation by specific inhibiting BACH1. This mechanism of action is related to the promotion of VEGF expression in endothelial cells and may also be related to increased energy metabolism, anti-inflammatory activity, anti-oxidant activity, or a combination of these functions. The specific mechanism needs to be further studied and verified in human primary endothelial cells and animal experiments.

## Conclusions

In summary, we have provided direct evidence of BACH1 expression in HMEC-1 cells and that 20 μM haem can inhibit BACH1 expression in HMEC-1 cells. Moreover, we demonstrated that 40% hyperoxia could inhibit the proliferation, migration and tube formation of HMEC-1 cells, and a moderate concentration of haem could relieve the hyperoxia-induced inhibition of angiogenesis.

## Supplementary Information

**Additional file 1.** The certificate of HMEC-1 cells from ATCC.

**Additional file 2.** The STR test result of HMEC-1 cells.

**Additional file 3 **Original Western blot images. **Fig. S1** Effect of haem on HRMEC cell proliferation. a, Diagram showing the effect of different concentrations of haem on HRMEC- cell proliferation as detected by the CCK-8 method. All data are presented as the means ± SD, and the experiment was repeated three times (*n* = 3). One-way ANOVA or multivariate ANOVA was performed. b, Diagram showing the effect of different incubation times of 20 μM haem on HRMEC cell proliferation as detected by the CCK-8 method. All data are presented as the mean ± SD, and the experiment was repeated three times (*n* = 3). Multivariate ANOVA with repeated measurements test was performed. c, Images representative of the proliferative activity of HRMEC cells as measured by the EdU assay. Under a laser confocal microscope, proliferating cells were stained green (488), and nuclei were stained blue (DAPI). The arrow indicates a representative HRMEC cell with proliferative activity (scale bar = 200 μm). d-f, Quantitative analysis of the total number of cells, the number of cells with proliferative activity and the percentage of cells with proliferative activity among HRMEC cells subjected to different treatments. All data are presented as the means ± SD, and the experiment was repeated three times (*n* = 3). One-way ANOVA or multivariate ANOVA was performed. **Fig. S2** Effect of haem on the angiogenesis of HRMEC cells. a, Images of the capillary-like tube formation of HRMEC cells stained green (488) after 48 h of culture under different conditions. Under hyperoxic conditions alone, the vascular network was fragmented and disordered. Upon the addition of 20 μM haem, the vascular network was more complete and denser (scale bar = 200 μM). b-d, Quantitative analysis of the number of junctions, length and area of capillary-like tubes formed by HRMEC cells. All data are presented as the means ± SD, and the experiment was repeated three times (*n* = 3). One-way ANOVA or multivariate ANOVA was performed.

## Data Availability

All data used to support the findings of this study are included in this article.

## Abbreviations

ATCC: American Type Culture Collection
BACH1: BTB and CNC homology l
CCK8: Cell counting kit 8
DAPI: 4′,6-diamidino-2-phenylindole
EdU: 5-ethynyl-2′-deoxyuridine
ETC: Electron transfer chain
FBS: Foetal bovine serum
HBSS: Hank’s balanced salt solution
HMEC-1: Human microvascular endothelial cells
HO-1: Haem degrading enzyme
HPLC: High-performance liquid chromatography
HRMECs: Human retinal microvascular endothelial cells
IF: Immunofluorescence
MEL: Murine erythroleukemia
NICU: Neonatal intensive care unit
PBS: Phosphate-buffered saline
PECAM-1: Platelet/endothelial cell adhesion molecule-1
RGC: Retinal ganglion cells
ROP: Retinopathy of prematurity
TCA: Tricarboxylic acid cycle
TNBC: Triple-negative breast cancer
VEGF: Vascular endothelial growth factor
