# Supplementary material for: Haem relieves hyperoxia-mediated inhibition of HMEC-1 cell proliferation, migration and angiogenesis by inhibiting BACH1 expression
Source: BMC Ophthalmol. 2021 Feb 25;21:104. doi: 10.1186/s12886-021-01866-x (PMC7905865; doi:10.1186/s12886-021-01866-x)

Sample File	Sample Name	Panel	SQO	SQ
hmec-1.4sk.fsa	hmec-1.4	20A_v1.6		

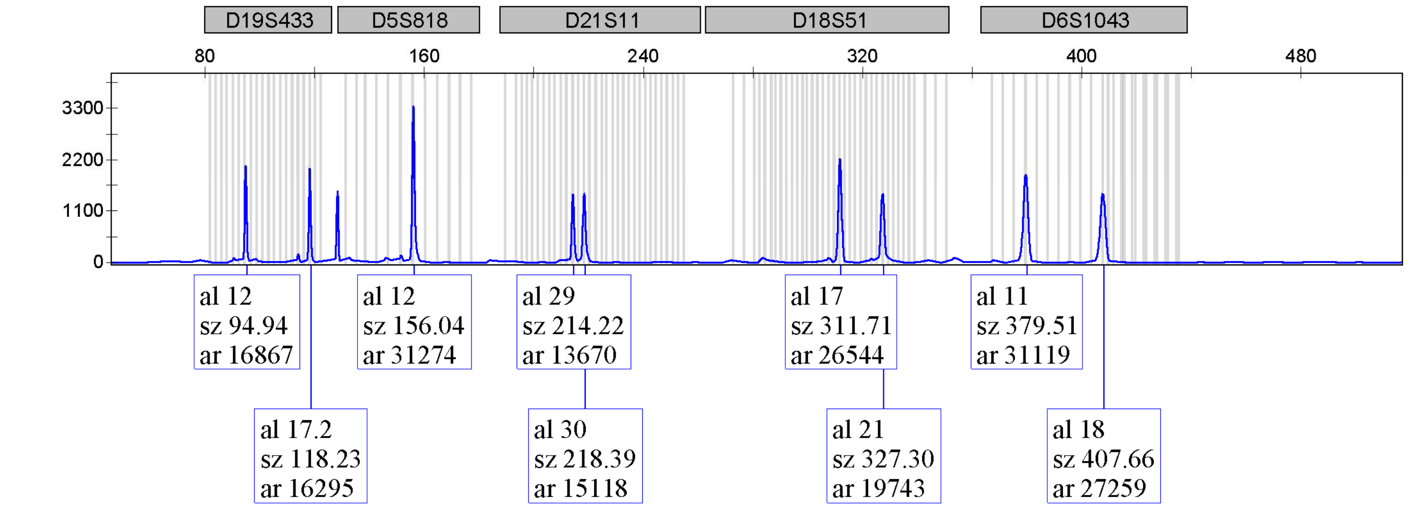

hmec-1.4sk.fsa	hmec-1.4	20A_v1.6		

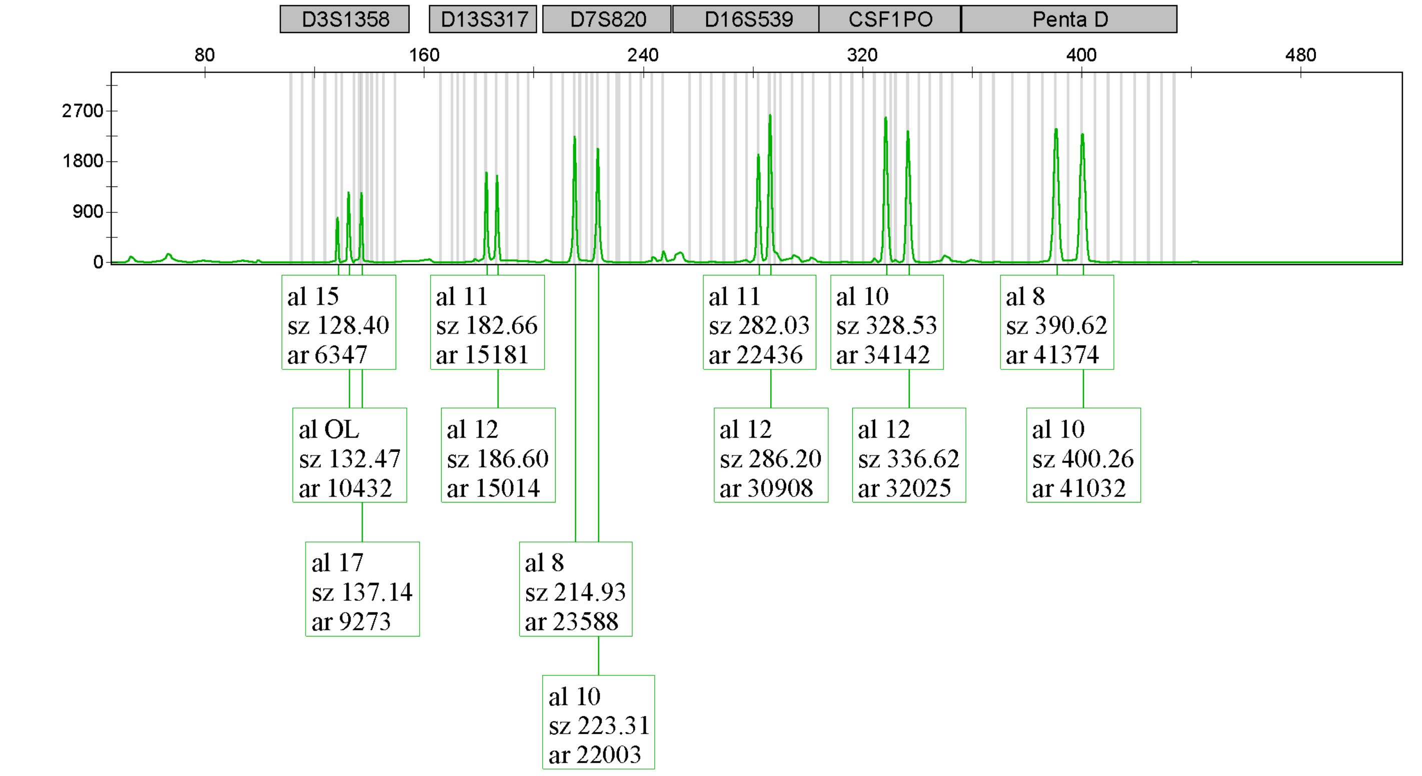

Sample File	Sample Name	Panel	SQO	SQ
hmec-1.4sk.fsa	hmec-1.4	20A_v1.6		

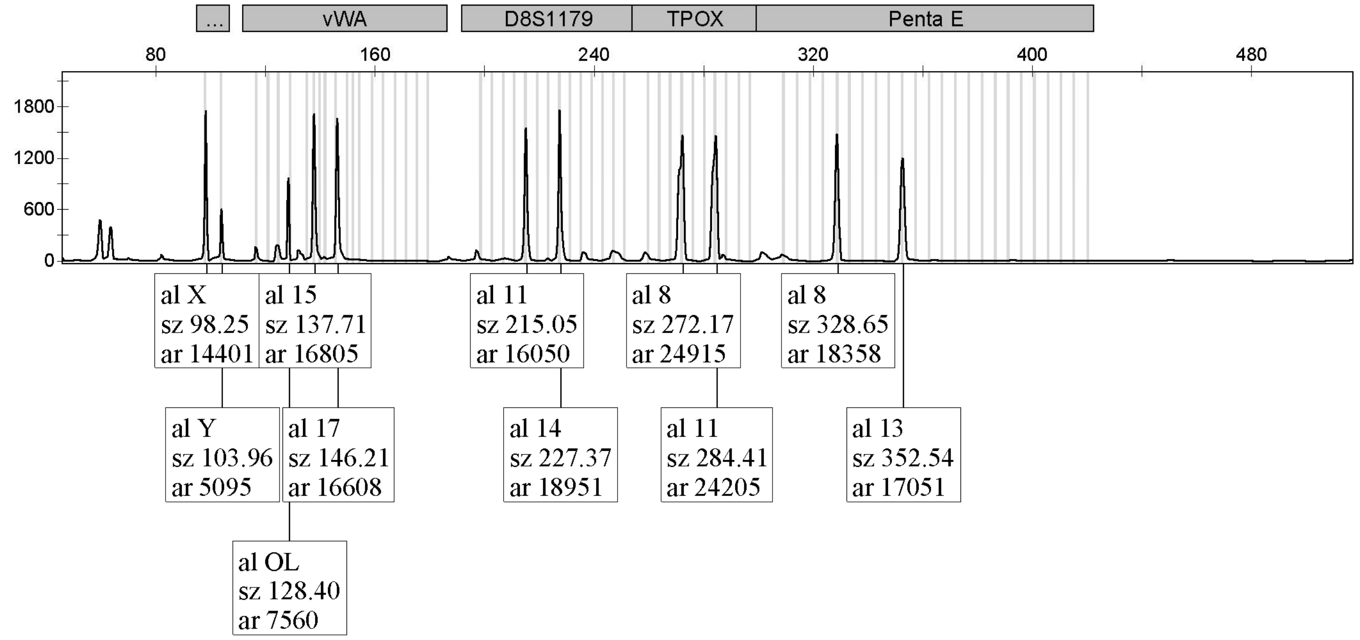

hmec-1.4sk.fsa	hmec-1.4	20A_v1.6		

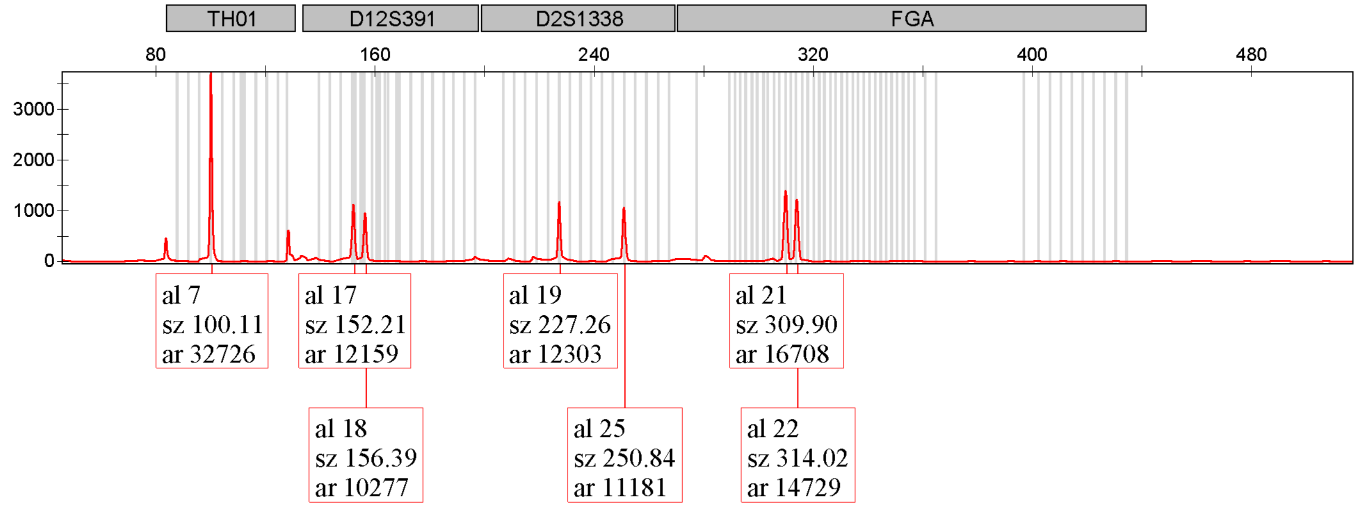

hmec-1.4sk.fsa	hmec-1.4	20A_v1.6		

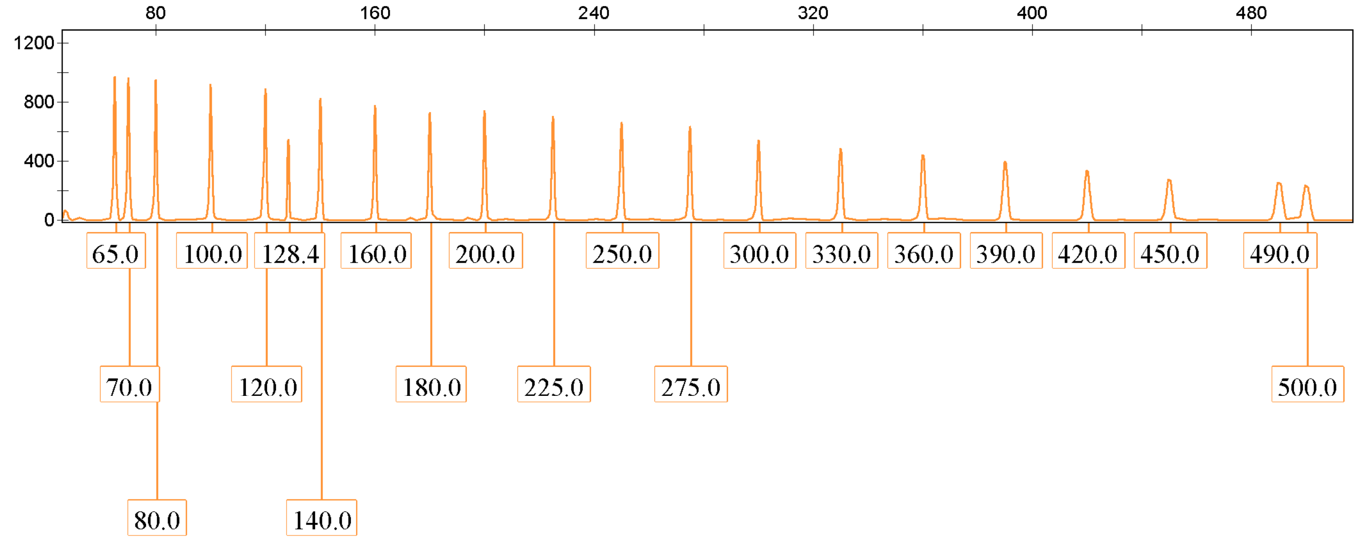

Supplement: Supplementary file 2 — Additional file 2. The STR test result of HMEC-1 cells. [file 12886_2021_1866_MOESM2_ESM.pdf]
