# Supplementary figures and images for: Haem relieves hyperoxia-mediated inhibition of HMEC-1 cell proliferation, migration and angiogenesis by inhibiting BACH1 expression
Source: BMC Ophthalmol. 2021 Feb 25;21:104. doi: 10.1186/s12886-021-01866-x (PMC7905865; doi:10.1186/s12886-021-01866-x)

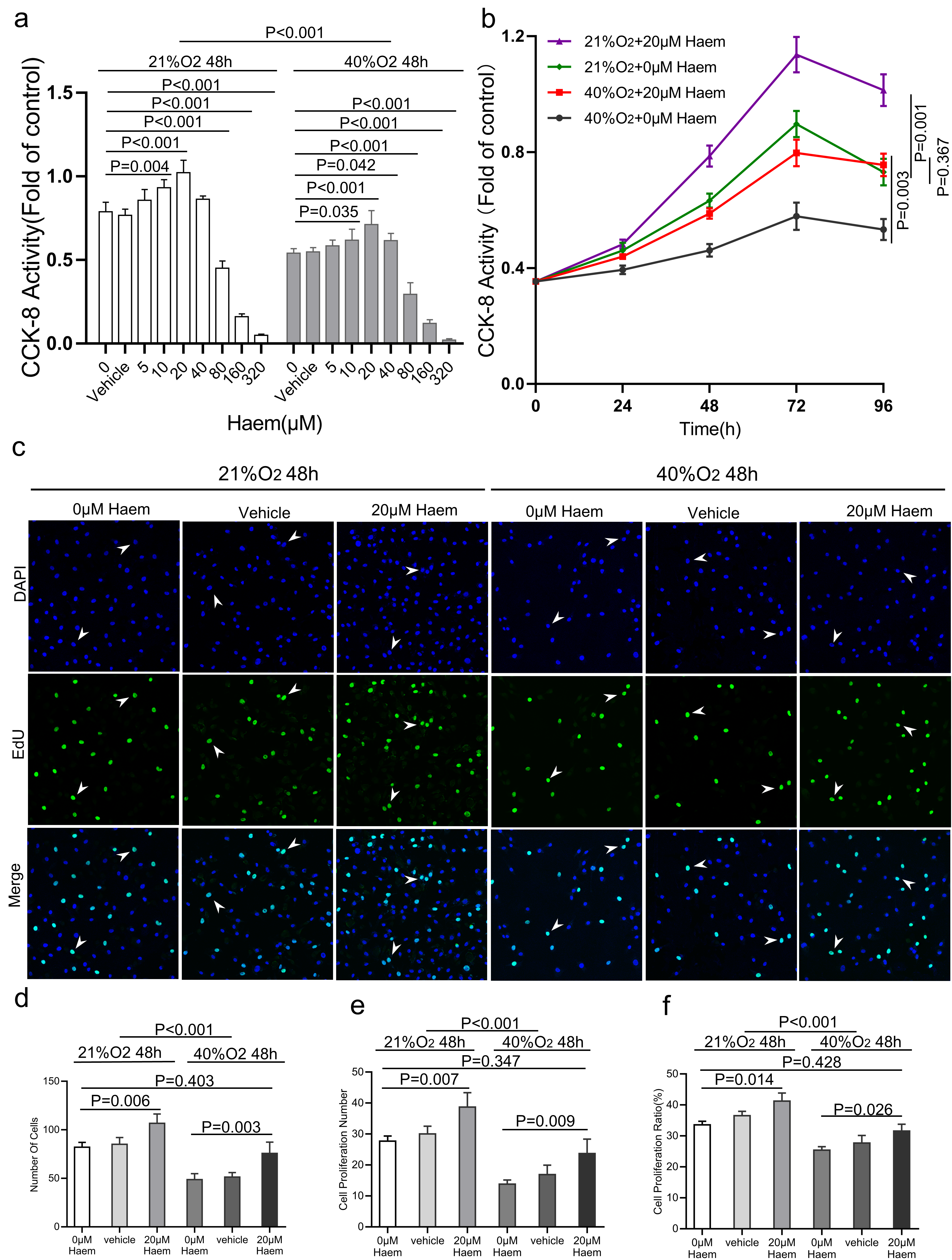

Supplement: Supplementary file 3 — Additional file 3 Original Western blot images. Fig. S1 Effect of haem on HRMEC cell proliferation. a, Diagram showing the effect of different concentrations of haem on HRMEC- cell proliferation as detected by the CCK-8 method. All data are presented as the means ± SD, and the experiment was repeated three times (n = 3). One-way ANOVA or multivariate ANOVA was performed. b, Diagram showing the effect of different incubation times of 20 μM haem on HRMEC cell proliferation as detected by the CCK-8 method. All data are presented as the mean ± SD, and the experiment was repeated three times (n = 3). Multivariate ANOVA with repeated measurements test was performed. c, Images representative of the proliferative activity of HRMEC cells as measured by the EdU assay. Under a laser confocal microscope, proliferating cells were stained green (488), and nuclei were stained blue (DAPI). The arrow indicates a representative HRMEC cell with proliferative activity (scale bar = 200 μm). d-f, Quantitative analysis of the total number of cells, the number of cells with proliferative activity and the percentage of cells with proliferative activity among HRMEC cells subjected to different treatments. All data are presented as the means ± SD, and the experiment was repeated three times (n = 3). One-way ANOVA or multivariate ANOVA was performed. Fig. S2 Effect of haem on the angiogenesis of HRMEC cells. a, Images of the capillary-like tube formation of HRMEC cells stained green (488) after 48 h of culture under different conditions. Under hyperoxic conditions alone, the vascular network was fragmented and disordered. Upon the addition of 20 μM haem, the vascular network was more complete and denser (scale bar = 200 μM). b-d, Quantitative analysis of the number of junctions, length and area of capillary-like tubes formed by HRMEC cells. All data are presented as the means ± SD, and the experiment was repeated three times (n = 3). One-way ANOVA or multivariate ANOVA was performed. [file 12886_2021_1866_MOESM3_ESM.zip › Additional Figure1R1.tif]

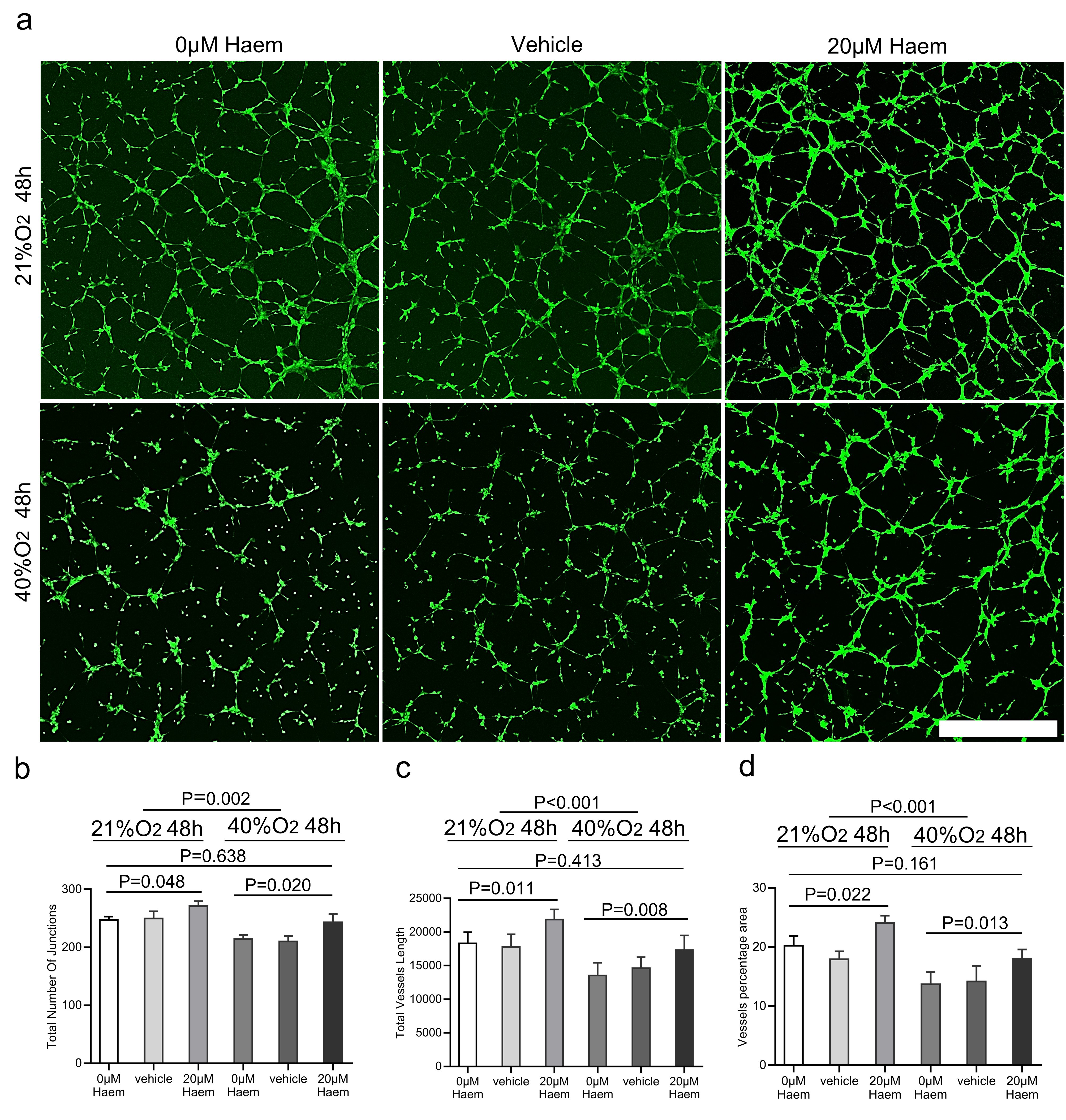

Supplement: Supplementary file 3 — Additional file 3 Original Western blot images. Fig. S1 Effect of haem on HRMEC cell proliferation. a, Diagram showing the effect of different concentrations of haem on HRMEC- cell proliferation as detected by the CCK-8 method. All data are presented as the means ± SD, and the experiment was repeated three times (n = 3). One-way ANOVA or multivariate ANOVA was performed. b, Diagram showing the effect of different incubation times of 20 μM haem on HRMEC cell proliferation as detected by the CCK-8 method. All data are presented as the mean ± SD, and the experiment was repeated three times (n = 3). Multivariate ANOVA with repeated measurements test was performed. c, Images representative of the proliferative activity of HRMEC cells as measured by the EdU assay. Under a laser confocal microscope, proliferating cells were stained green (488), and nuclei were stained blue (DAPI). The arrow indicates a representative HRMEC cell with proliferative activity (scale bar = 200 μm). d-f, Quantitative analysis of the total number of cells, the number of cells with proliferative activity and the percentage of cells with proliferative activity among HRMEC cells subjected to different treatments. All data are presented as the means ± SD, and the experiment was repeated three times (n = 3). One-way ANOVA or multivariate ANOVA was performed. Fig. S2 Effect of haem on the angiogenesis of HRMEC cells. a, Images of the capillary-like tube formation of HRMEC cells stained green (488) after 48 h of culture under different conditions. Under hyperoxic conditions alone, the vascular network was fragmented and disordered. Upon the addition of 20 μM haem, the vascular network was more complete and denser (scale bar = 200 μM). b-d, Quantitative analysis of the number of junctions, length and area of capillary-like tubes formed by HRMEC cells. All data are presented as the means ± SD, and the experiment was repeated three times (n = 3). One-way ANOVA or multivariate ANOVA was performed. [file 12886_2021_1866_MOESM3_ESM.zip › Additional Figure2R1.tif]
